# Buformin inhibits the stemness of erbB-2-overexpressing breast cancer cells and premalignant mammary tissues of MMTV-erbB-2 transgenic mice

**DOI:** 10.1186/s13046-017-0498-0

**Published:** 2017-02-13

**Authors:** Amanda B. Parris, Qingxia Zhao, Erin W. Howard, Ming Zhao, Zhikun Ma, Xiaohe Yang

**Affiliations:** 1 Julius L. Chambers Biomedical/Biotechnology Research Institute, Department of Biological and Biomedical Sciences, North Carolina Central University, 500 Laureate Way, NRI 4301, Kannapolis, North Carolina 28081 USA; 2 College of Medicine, Henan University of Sciences and Technology, Luoyang, China

**Keywords:** Buformin, Cancer stem cells, ErbB-2, Mammary epithelial cells, Breast cancer

## Abstract

**Background:**

Metformin, an FDA-approved drug for the treatment of Type II diabetes, has emerged as a promising anti-cancer agent. Other biguanide analogs, including buformin and phenformin, are suggested to have similar properties. Although buformin was shown to reduce mammary tumor burden in carcinogen models, the anti-cancer effects of buformin on different breast cancer subtypes and the underlying mechanisms remain unclear. Therefore, we aimed to investigate the effects of buformin on erbB-2-overexpressing breast cancer with *in vitro* and *in vivo* models.

**Methods:**

MTT, cell cycle, clonogenic/CFC, ALDEFLUOR, tumorsphere, and Western blot analyses were used to determine the effects of buformin on cell growth, stem cell populations, stem cell-like properties, and signaling pathways in SKBR3 and BT474 erbB-2-overexpressing breast cancer cell lines. A syngeneic tumor cell transplantation model inoculating MMTV-erbB-2 mice with 78617 mouse mammary tumor cells was used to study the effects of buformin (1.2 g buformin/kg chow) on tumor growth *in vivo*. MMTV-erbB-2 mice were also fed buformin for 10 weeks, followed by analysis of premalignant mammary tissues for changes in morphological development, mammary epithelial cell (MEC) populations, and signaling pathways.

**Results:**

Buformin significantly inhibited SKBR3 and BT474 cell growth, and *in vivo* activity was demonstrated by considerable growth inhibition of syngeneic tumors derived from MMTV-erbB-2 mice. In particular, buformin suppressed stem cell populations and self-renewal *in vitro*, which was associated with inhibited receptor tyrosine kinase (RTK) and mTOR signaling. Consistent with *in vitro* data, buformin suppressed mammary morphogenesis and reduced cell proliferation in MMTV-erbB-2 mice. Importantly, buformin decreased MEC populations enriched with mammary reconstitution units (MRUs) and tumor-initiating cells (TICs) from MMTV-erbB-2 mice, as supported by impaired clonogenic and mammosphere formation in primary MECs. We further demonstrated that buformin-mediated *in vivo* inhibition of MEC stemness is associated with suppressed activation of mTOR, RTK, ER, and β-catenin signaling pathways.

**Conclusions:**

Overall, our results provide evidence for buformin as an effective anti-cancer drug that selectively targets TICs, and present a novel prevention and/or treatment strategy for patients who are genetically predisposed to erbB-2-overexpressing breast cancer.

**Electronic supplementary material:**

The online version of this article (doi:10.1186/s13046-017-0498-0) contains supplementary material, which is available to authorized users.

## Background

Breast cancer prevention and treatment are often met with significant challenges due to the heterogeneity of mammary tumors that contribute to poor prognosis. For instance, cancer preventatives, like tamoxifen for estrogen receptor-positive (ER^+^) breast cancers, have shown clinical efficacy; yet, the prevention of ER- breast cancers, including the erbB-2-overexpressing subtype, remains elusive [1, 2]. Consequently, the search for novel strategies to prevent and treat breast cancer has expanded over the past decade to drugs that have shown promise in other disease models and other cancer sites. In particular, metformin, a biguanide drug commonly prescribed to treat Type II diabetes in humans, has demonstrated anti-cancer effects, which was published in an earlier milestone report by Evans *et al.* (2005) showing that metformin significantly reduced the risk of developing multiple types of cancer in patients with diabetes [3]. Thereafter, other studies corroborated that metformin lowered breast cancer risk [4]. Breast cancers, including the erbB-2-overexpressing subtype, are often associated with morbidity and poor clinical outcomes; therefore, the identification and development of effective erbB-2-overexpressing breast cancer prevention and treatment options are crucial [5–7].

In regard to the need for breast cancer preventatives, many preclinical studies and clinical trials have been initiated to determine the underlying mechanisms involved in the reported anti-cancer effects of metformin and to develop metformin as a novel breast cancer preventative strategy by optimizing treatment doses and conditions. As such, preclinical studies have explored the preventative effects of metformin in various cell and animal models of cancer. In prostate cancer LNCaP and PC-3 cell lines, metformin has been shown to induce anti-cancer effects through the inhibition of cell growth and the activation of AMPK-dependent and MAPK-mediated apoptosis [8–10]. Similarly, metformin has previously been reported to induce MAPK-mediated apoptosis in addition to GADD153-mediated apoptosis in A549 and NCI-H1299 human lung cancer cell lines [11]. Breast cancer has also been the focus of many studies determining the efficacy and underlying mechanism of the cancer preventative activities of metformin. In breast cancer cell lines, including MCF-7, MCF-10A, and MDA-MB-231 (p53 wt) cells, metformin stimulated the inhibition of cell proliferation and the induction of apoptosis, which were found to be dependent on AMPK and p53 status in the cells [8, 12]. As such, data from our lab have previously reported that metformin can significantly inhibit growth of syngeneic erbB-2-overexpressing mammary tumors from MMTV-erbB-2 transgenic mice inoculated with 78617 cells [13]. Results from preclinical cell and animals models testing the anti-cancer effects of metformin are further reflected in clinical trials. A meta-analysis of 11 clinical studies testing the anti-cancer effects of metformin determined that metformin reduced colon, prostate, and breast cancer risk by up to 31%, collectively [14].

Although metformin has demonstrated substantial clinical cancer protective benefits, the mechanisms behind the anti-cancer properties of metformin are not completely understood. As a Type II diabetes therapeutic, metformin disrupts the Warburg effect and reduces glucose output by the liver and circulating insulin levels [15]. These effects on glucose metabolism are also demonstrated in non-diabetic models alongside a characteristic upregulation of the energy sensor AMPK, through the inhibition of the mitochondrial complex I [4, 16–18]. The activation/phosphorylation of AMPK subsequently inhibits proliferative cellular responses associated with the mTOR signaling pathway [4, 18]. In particular, metformin blocked mTOR-dependent translation, which is essential for the regulation of cell growth, survival, and angiogenesis, in MCF-7 breast cancer cells [19, 20]. Alternatively, Ben Sahra *et al.* (2011) reported that metformin can induce AMPK-independent cellular responses under hypoxic conditions in LNCaP prostate cancer cells, including REDD1-mediated mTOR inhibition and subsequent cell cycle arrest [21]. Moreover, the effects of metformin on glucose metabolism and mTOR signaling inhibition are also implicated in cancer stem cell (CSC) regulation as previously shown in syngeneic tumor, xenograft tumor, and transgenic mouse models of breast cancer [13, 22, 23]. CSCs contribute to tumor development because of increased proliferative and self-renewal capabilities [24]. Metformin further inhibits cancer cell proliferation through blockage of the IGF/IGF-1R signaling pathway, as shown in PC-3 prostate cancer cells, MKN1, MKN45, and MKN74 gastric cancer cells, and SKBR3 and BT474 breast cancer cells [25–27].

Based on the presented studies showing the anti-cancer effects of metformin across a range of cell, animal, and human cancer models, metformin is a promising cancer preventative drug. Nevertheless, several challenges still remain. To this end, the specific mechanisms of action, clinical efficacy, and exploration of metformin analogs warrants further investigation. In regards to analogs with structural and functional similarities to metformin, recent studies using phenformin and buformin indicate increased bioactivity and anti-cancer effects than metformin [28–30]. Similar to metformin, buformin also has demonstrated anti-cancer properties through the induction of cell cycle arrest, apoptosis, and reduced cell adhesion and migration in ECC-1 and Ishikawa endometrial cell lines [31]. In these cells, buformin exhibited a lower IC_50_ value than metformin as well. Furthermore, Zhu *et al.* (2015) revealed that buformin (7.6 mM/kg) decreased tumor incidence and burden to a greater extent than metformin (9.3 mM/kg) and phenformin (5 mM/kg) in a 1-methyl-1-nitrosourea (MNU)-induced mammary carcinogenesis model using Sprague–Dawley rats [30]. Despite buformin being withdrawn from the market due to tolerance concerns, these promising reports detailing the anti-cancer properties of buformin *in vitro* and *in vivo* indicate that further studies into the efficacy and mechanisms of buformin are necessary in order to determine a dose that will maintain anti-cancer benefits while increasing drug tolerance.

Our lab recently studied the cancer preventative effects of metformin treatment in cell and animal models of erbB-2-overexpressing breast cancer [13]. We found that metformin significantly inhibited cell proliferation and the stemness of erbB-2-overexpressing breast cancer cell lines. Likewise, metformin inhibited tumor growth in MMTV-erbB-2 transgenic mice through the targeting of the CSCs [13]. Due to the limited number of studies on the anti-cancer efficacy of buformin, the effects on specific, refractory breast cancer subtypes, like erbB-2-positive breast cancer, remains to be explored. To this end, using the well-established MMTV-erbB-2 mouse model in our current study, we found that buformin inhibits cell proliferation, cell cycle, and CSC self-renewal properties in erbB-2-overexpressing breast cancer cells. These findings also translated to a syngeneic tumor transplantation model in MMTV-erbB-2 mice that indicated buformin, when administered in the diet, inhibited tumor growth. Furthermore, we demonstrated that buformin diet produced significant inhibition of mammary morphogenesis and CSC populations in premalignant mammary tissues from MMTV-erbB-2 mice. Taken together, our data provide evidence for buformin as an effective anti-cancer drug, especially in patients at a high risk for developing erbB-2-overexpressing breast cancers, and may ultimately have a significant impact on breast cancer prevention.

## Methods

### Antibodies and reagents

Buformin was purchased from Wako Pure Chemical Industries (Osaka, Japan). Primary antibodies against AMPK, p-AMPK, mTOR, p-mTOR, p70S6K, p-p70S6K, 4EBP1, p-4EBP1, IRS, p-IRS, IGF1R, p-IGF1R, p-erbB-2, Akt, p-Akt, p-Erk1/2, p-Stat3, p-ER, β-catenin, Oct4A, and Notch were purchased from Cell Signaling (Danvers, MA). Antibodies against IGF1Rα/β, Erk, Stat3, ER, Cyclin D1, and β-actin were ordered from Santa Cruz Biotechnology (Santa Cruz, CA). erbB-2 and active β-catenin antibodies were purchased from EMD Millipore (Billerica, CA).

### Cell culture and treatment

Breast cancer cell lines used in these studies, including SKBR3 and BT474 cells from ATCC (Manassas, VA) and mammary tumor-derived 78617 cells, were maintained in DMEM/F-12 culture medium supplemented with 10% fetal bovine serum (FBS), 100 μg/ml penicillin, and 100 μg/ml streptomycin at 37 °C in an incubator with a humidified 5% CO_2_ atmosphere. The medium was replaced and cells were treated with buformin 24 h after seeding. The 78617 breast cancer cell line was established in our lab from FVB/N-Tg/MMTV-erbB-2 (MMTV-erbB-2) transgenic mouse mammary tumors as previously reported [32].

### Cell viability assay

SKBR3 and BT474 cells were plated (1 × 10^3^ cells/well) in 96-well plates for 24 h. Then the cells were incubated in indicated doses of buformin for 5 days. Following drug treatment, the cells were incubated at 37 °C for 4 h in 3-(4,5-dimethythiazol-2-yl)-2,5-diphenyltetrazolium bromide (MTT). The medium was removed and 50 μl DMSO was added to each well and incubated at room temperature for 45 min while shaking. Absorbance was measured using a SynergyMx microplate reader (BioTek; Winooski, VT) to determine the viable cell fraction.

### Cell cycle analysis

SKBR3 and BT474 cells were treated with indicated concentrations of buformin for 48 h. Then, the cells were trypsinized and fixed in ice-cold 70% ethanol (added drop-wise) at −20 °C overnight. Fixed cells were centrifuged to form a cell pellet and the supernatant was removed. The cells were resuspended and incubated in PBS containing 0.2% triton X-100, 500 μg/ml RNase A, and 33 μg/ml propidium iodide at 37 °C for 45 min. Finally, the number of cells in each phase of the cell cycle was analyzed using fluorescence-activated cell sorting (FACS) and ModFit software.

### Clonogenic and colony-forming cell (CFC) assays

SKBR3 and BT474 cells were seeded at 1 × 10^3^ and 2 × 10^3^ cells/well, respectively, in 6-well plates for 24 h and then treated with indicated doses of buformin for 14 days. The isolated primary mammary epithelial cells (MECs) from control-fed and buformin-fed mice were seeded at 4 × 10^3^ cells/plate in 60 mm plates and incubated for 7 days. After incubation, the cultured cells were washed with PBS and stained with 0.5% crystal violet (1:1 of methanol:H_2_O). The primary cells were washed with PBS, fixed with acetone:methanol (1:1), and stained with Wright’s Giemsa. Stained colonies were imaged using the Nikon SMZ 745 T microscope and Nikon Elements Imaging System Software. The number of colonies with ≥ 50 cells was recorded for each sample. All treatments were tested in at least triplicate.

### Animals and buformin diet

Female MMTV-erbB-2 transgenic mice were purchased from Jackson Laboratories (Bar Harbor, ME). All mice were fed a standard, estrogen-free AIN-93G diet (Bio-Serv; Flemington, NJ) until 8 weeks of age when the mice were divided into control and experimental groups. The control mice continued receiving the AIN-93G diets, while the treatment group received an AIN-93G-based buformin diet that contained 1.2 g of buformin/kg of chow (7.6 mmol buformin/kg of chow) [30]. Based on the assumptions that a mouse weighing 25 g will eat approximately 6 g of chow/day, we estimate that each mouse being fed the buformin diet will consume 7.2 mg of buformin/day. The mice were fed control and buformin diets for 10 weeks until the mice reached 18 weeks of age and were euthanized for further analysis of collected tissues. All animal procedures were performed according to IACUC-approved protocols.

### Syngeneic tumor model

8-week-old MMTV-erbB-2 mice were inoculated with a subcutaneous injection of 78617 cells (5 × 10^5^) in each flank. By 6 days after inoculation with the tumor cells, palpable tumors were formed in the mice and half of the mice were fed the buformin diet described above, while the remaining mice continued on the standard AIN-93G diet. Tumors were palpated every 2 days beginning 6 days after inoculation with the tumor cells and tumor volumes were calculated as: Volume (mm^3^) = (width^2^ × length)/2. On Day 18 after the initial tumor cell inoculation, the mice were euthanized and the tumors were excised, weighed, and imaged.

### ALDEFLUOR assay

The ALDEFLUOR kit (Stemcell Technologies; Cambridge, MA) was used to measure aldehyde dehydrogenase 1 (ALDH1) activity in breast cancer cell lines. Cells were treated with indicated doses of buformin for 48 h followed by incubation with the ALDEFLUOR substrate at 37 °C for 30 min. To define the ALDEFLUOR-positive region, the ALDH1 inhibitor, diethylaminobenzaldehyde (DEAB), was used for negative control samples. Four replicates of control and treated samples were analyzed with flow cytometry using Guava EasyCyte Flow Cytometer (EMD Millipore) and FlowJo analysis software to determine the percentage of ALDH-positive (ALDH^+^) cells.

### Tumorsphere and mammosphere assays

For tumorsphere assays, 78617 or BT474 cells were seeded (800 cells/well) in ultra-low attachment 24-well plates (Corning). For mammosphere assays, primary MECs were plated (2.5 × 10^4^ cells/well) in ultra-low attachment 24-well pates. The cell lines (treated with indicated doses of buformin) and primary MECs were incubated in DMEM/F-12 medium and EpiCult-B Mouse Media (Stemcell Technologies), respectively, supplemented with 10 μg/ml insulin (Sigma; St. Louis, MO), 1 μg/ml hydrocortisone (Sigma), 1x B-27 (Thermo Fisher Scientific), 20 ng/ml EGF (Stemcell Technologies), 20 ng/ml bFGF (Stemcell Technologies), 4 μg/ml heparin (Stemcell Technologies), and 50 μg/ml Gentamycin for 7 days to form primary spheres. After 7 days, primary spheres between 40 – 120 μm in diameter were counted and imaged before trypsinization. The harvested primary spheres were pipetted to form a single cell suspension and were then replated using the same conditions to form secondary spheres. Secondary spheres that formed after 7 days of incubation were counted and imaged for analysis. Primary and secondary sphere assays were performed in at least triplicates.

### Western blot analysis

Whole cell protein lysates were collected from breast cancer cell lines or homogenized mammary tissues from 18-week-old MMTV-erbB-2 mice. Protein concentrations were determined using a BCA Protein assay kit (Thermo Fisher Scientific). Fifty μg of protein were loaded in 10 or 12% SDS-PAGE gels. Proteins were separated using gel electrophoresis and were then transferred to nitrocellulose membranes. Membranes were blocked in 5% milk for 2 h at room temperature and then incubated in primary antibodies at 4 °C overnight. After washing, the membranes were incubated in secondary horseradish peroxidase-labeled antibodies for 1 h at room temperature. Protein bands were visualized using SuperSignal West Pico Chemiluminescent solution (Thermo Fisher Scientific) and imaged using a FluorChemE imager.

### Whole mount analysis

Mammary glands were excised from 18-week-old MMTV-erbB-2 mice and were placed on glass slides for fixation in Carnoy’s solution at room temperature overnight. The mammary glands were rehydrated with decreasing concentrations of ethanol for 30 min, followed by overnight staining in carmine alum stain. The next day, the stained glands were dehydrated with increasing concentrations of ethanol, cleared with xylene, and mounted using Permount (Thermo Fisher Scientific). The mounted mammary glands were imaged using a Nikon Eclipse 80i microscope and the Nikon Elements Imaging System (Nikon Instruments, Inc.).

### Histology and immunohistochemistry (IHC)

For histological and IHC analyses, formalin-fixed, paraffin-embedded (FFPE) mammary glands from 18-week-old MMTV-erbB-2 mice were deparaffinized and rehydrated with xylene and ethanol, respectively. Then, for hematoxylin and eosin (H&E) staining, the tissues were stained with hematoxylin for 5 min, followed by separate water, acid alcohol (1% HCl in ethanol), and 0.2% ammonia washes. After the consecutive washes, the FFPE tissues were stained with eosin Y for 1 min. Before mounting the stained tissues with Permount, the tissues were dehydrated and cleared.

For IHC, the FFPE slides were boiled in citrate buffer (pH 6.0) for 30 min at 100 °C for antigen retrieval. Then the slides were blocked in 3% H_2_O_2_ in methanol for 10 min at room temperature. Nonspecific binding was blocked using 10% horse serum, followed by overnight incubation in the Ki67, p-mTOR, mTOR, or p-AMPK primary antibodies at 4 °C. After the overnight incubation in the primary antibody, the slides were incubated in secondary antibody for 1 h at room temperature. The ABC reagent (Vector Laboratories; Burlingame, CA) and diaminobenzidine (DAB) were used for color development reactions in the tissues, followed by counterstaining with hematoxylin and mounting for observational analyses. The Nikon Eclipse 80i microscope and Nikon Elements Imaging System Software were used to capture images of the stained sections.

### Primary MEC isolation

Mammary glands were excised from 18-week-old MMTV-erbB-2 mice and were homogenized using a tissue chopper (Mickle Laboratory Engineering). The dissociated tissues were digested in collagenase (Roche) and hyaluronidase (Sigma) at 37 °C for 2 h followed by digestion with 0.25% trypsin-EDTA (Sigma) and dispase (Roche)/DNase I (Sigma). The cell suspension was then filtered through a 40 μm mesh strainer, resulting in a single cell suspension for use in the described assays [33].

### Flow cytometry analysis

Isolated primary MECs were prepared for flow cytometry analysis using primary fluorescent antibodies against CD24, CD61, CD49f, and lineage markers according to the protocol used by Shelton *et al.* (2010) [34]. Cell populations were gated and analyzed using FlowJo analysis software.

### Statistical analysis

Statistical differences between two groups were determined using a Student’s *t*-test. A *p*-value of ≤ 0.05 was chosen for significance in all experiments.

## Results

### Buformin inhibits cell proliferation and induces cell cycle arrest in erbB-2-overexpressing breast cancer cells *in vitro*

**Fig. 1:**
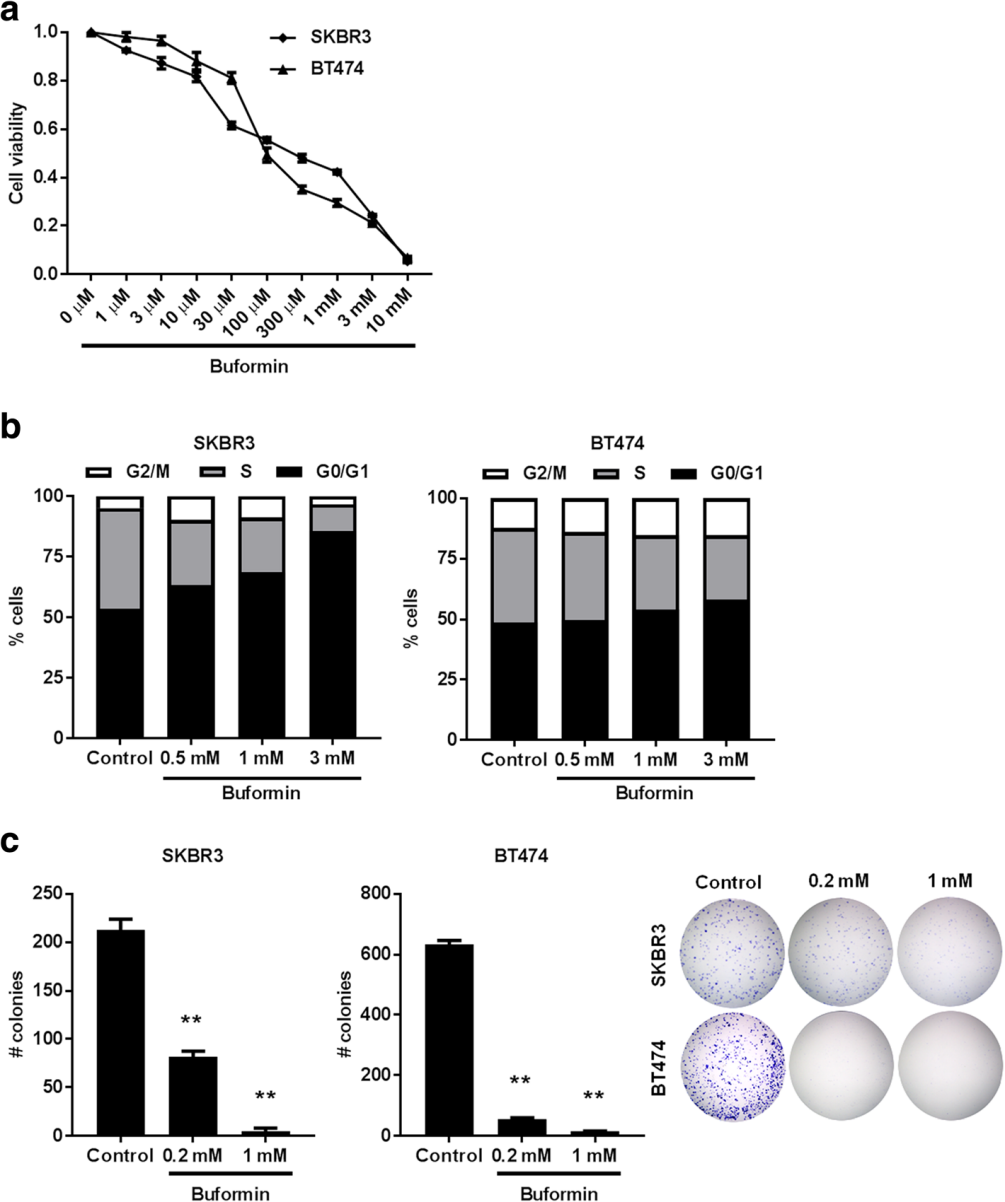
Buformin induces anti-proliferative effects in erbB-2-overexpressing SKBR3 and BT474 cells. **a** An MTT assay was performed to compare cell viability in SKBR3 and BT474 cells treated with buformin (0, 1, 3, 10, 30, 100, 300 μM, 1, 3, or 10 mM) for 5 days. The average viable cell fraction for each sample (N = 8) is plotted ± standard error (S.E.). **b** SKBR3 and BT474 cells were treated with buformin (0, 0.5, 1, or 3 mM) for 48 h and then cell cycle progression was measured using FACS analysis (N = 4). The average percentage of cells in G2/M, S, and G0/G1 phases of the cell cycle are graphed for SKBR3 (left panel) and BT474 (right panel) cells. **c** SKBR3 and BT474 cells were treated with buformin (0, 0.2, or 1 mM) in triplicate for 14 days as part of the clonogenic assay. Then the colonies were fixed and stained with crystal violet. The average number of colonies that formed after 14 days is graphed. Representative images of crystal violet-stained colonies are shown in the far right panel. Values are graphed as the mean ± S.E. (**p < 0.01)

Metformin has demonstrated the capability to inhibit cell proliferation in various cancer cells, yet previous reports have shown that phenformin and buformin have increased biological activity than metformin [31]. We found that buformin reduced cell viability in erbB-2-overexpressing SKBR3 (IC_50_ = 246.7 μM) and BT474 (IC_50_ = 98.6 μM) breast cancer cell lines (Fig. 1a). As shown in Fig. 1b, further investigation into the cellular responses caused by buformin revealed buformin-induced G0/G1 cell cycle arrest in SKBR3 and BT474 breast cancer cells. In both cell lines, buformin increased the percentage of cells in G0/G1 phase and reduced the percentage of cells in S phase, especially in the SKBR3 cells, which is indicative of diminished proliferating cells. Impaired cell proliferation was also confirmed in both cell lines, as demonstrated by the reduction in the number of colonies formed in the clonogenic assay (Fig. 1c). Together, these data provide critical proof of concept in which buformin exhibits cell growth inhibitory effects *in vitro*.

### Buformin diet inhibits mammary syngeneic tumor growth in MMTV-erbB-2 transgenic mice

**Fig. 2:**
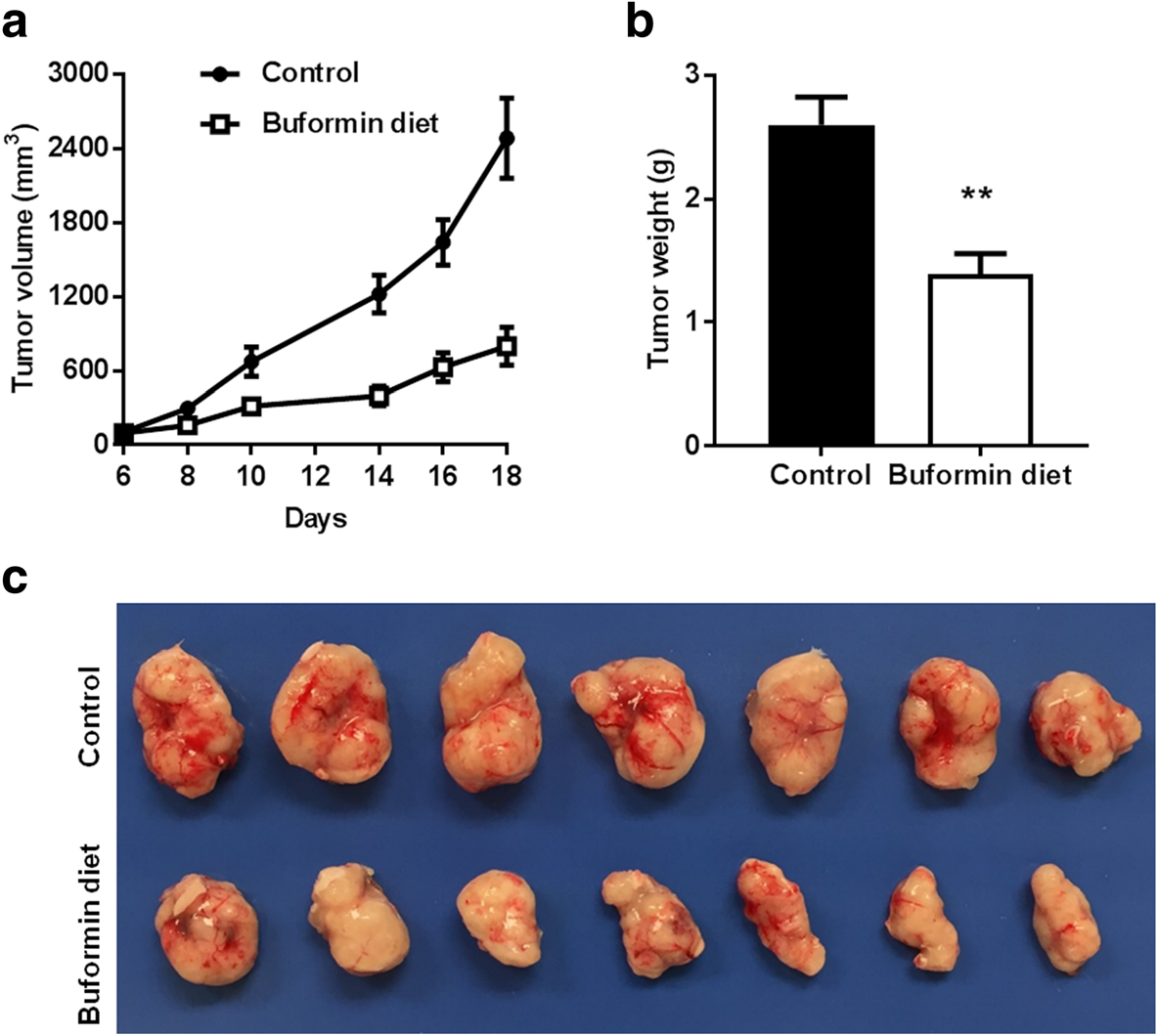
Buformin diet inhibits syngeneic tumor growth in MMTV-erbB-2 mice. 8-week-old MMTV-erbB-2 mice were inoculated with 78617 cells and tumors began to appear by Day 6 after the initial inoculation. At Day 6, mice (N = 7) were administered a buformin diet for 12 days with tumor palpitation every 2 days. The tumor volumes during the monitoring from Day 6 – 18 (**a**) and final tumor weights (**b**) were recorded. Representative images of tumors from the control and buformin-fed mice are shown in **c**. Values are graphed as the mean ± S.E. (**p < 0.01)

To further test the buformin-associated inhibitory effects *in vivo*, we used a syngeneic tumor model in MMTV-erbB-2 mice that were fed a buformin diet (1.2 g buformin/kg chow) for 12 days after inoculation with 78617 breast cancer cells. Mice fed the buformin diet exhibited significantly reduced tumor volumes and weights as compared to the control mice (Fig. 2). These *in vivo* results are consistent with our *in vitro* data demonstrating the anti-cancer potential of buformin.

### Buformin suppresses the stemness of erbB-2-overexpressing breast cancer cells *in vitro*

**Fig. 3:**
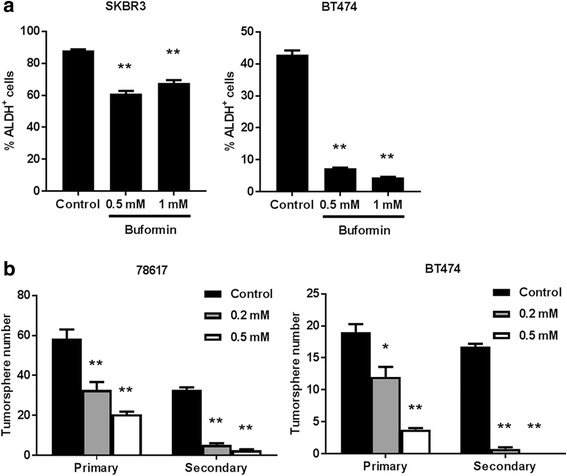
Buformin suppresses the stemness of breast cancer cells *in vitro*. **a** SKBR3 and BT474 cells were treated with buformin (0, 0.5, or 1 mM) for 48 h (N = 4). Then ALDH activity was measured using an ALDEFLUOR assay. The percentage of ALDH^+^ cells are presented in the graphs. **b** 78617 and BT474 cells were plated in ultra-low attachment plates and treated with buformin (0, 0.2, or 0.5 mM) for 7 days (N = 4). Primary tumorspheres were counted and imaged after 7 days. Then the cells were harvested and homogenized to form a single cell suspension that was replated in ultra-low attachment plates for another 7 days. Secondary tumorspheres were then counted and imaged. All values are graphed as the mean ± S.E. (*p < 0.05, **p < 0.01)

Increasing evidence supports a critical role of CSCs in cancer development [35, 36]. Our lab has previously determined that metformin targets CSC populations; therefore, we tested the effects of buformin on the CSC population using an ALDEFLUOR assay that detects ALDH activity, which is a characteristic of CSCs [13, 37]. In Fig. 3a, we show that buformin significantly lowers the percentage of ALDH^+^ SKBR3 and BT474 cells. Similarly, buformin inhibited tumorsphere formation, especially of secondary spheres of 78617, an erbB-2-overexpressing mammary tumor-derived cell line, and BT474 cells, indicating its inhibitory activity on CSC self-renewal (Fig. 3b). Altogether, these results suggest that the regulation of the CSC population and associated proliferative characteristics are implicated in the anti-proliferative/anti-cancer mechanism of buformin.

### Buformin regulates AMPK/mTOR and RTK signaling pathways in erbB-2-overexpressing breast cancer cells

**Fig. 4:**
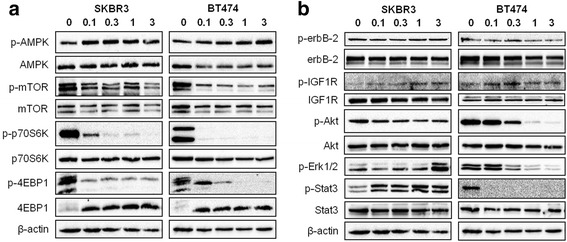
Buformin regulates AMPK/mTOR and RTK signaling pathways in erbB-2-overexpressing breast cancer cells. SKBR3 and BT474 cells were treated with buformin (0, 0.1, 0.3, 1, or 3 mM) for 24 h. Expression and activation/phosphorylation of the indicated proteins relating to the AMPK/mTOR (**a**) and RTK (**b**) pathways were analyzed using Western blotting

In order to understand which signaling pathways are regulated by buformin treatment and which may contribute to the CSC population changes that we have demonstrated, we performed Western blot analysis on SKBR3 and BT474 cells that were treated with low-dose (0 – 3 mM) buformin. Indeed, buformin treatment stimulated typical AMPK activation/phosphorylation that resulted in reduced mTOR activation/phosphorylation and remarkable inactivation of downstream signaling molecules, including p70S6K and 4EBP1 (Fig. 4a). Buformin also suppressed RTK activation, including erbB-2 and IGF1R signaling (Fig. 4b). Likewise, downstream Akt activation/phosphorylation was inhibited by buformin exposure in both cell lines. To note, SKBR3 and BT474 cells exhibited differential buformin-induced expression of phospho-Erk1/2 and phospho-Stat3, such that their expression was upregulated and downregulated in SKBR3 and BT474 cells, respectively. This observation will be further addressed in the *Discussion* section.

### Buformin diet during the premalignant risk window hinders mammary morphogenesis and proliferation in MMTV-erbB-2 transgenic mice

**Fig. 5:**
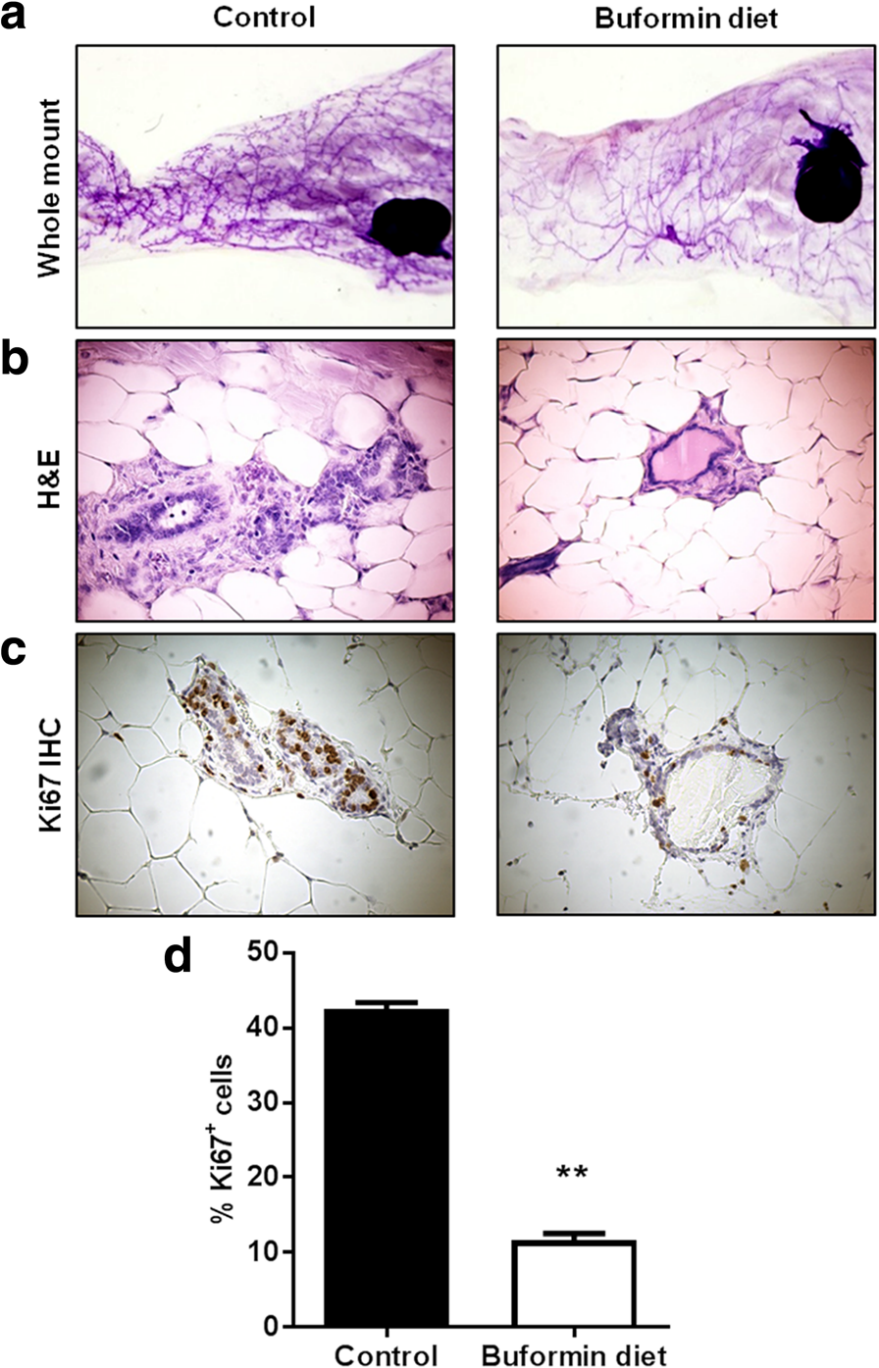
Buformin diet during the premalignant risk window hinders mammary morphogenesis and proliferation in MMTV-erbB-2 mice. Mice were fed control and buformin diets for 10 weeks (N = 6). At 18 weeks of age, the mice were sacrificed for whole mount preparation of mammary glands (**a**). Representative images of H&E stained (**b**) and Ki67 immunostained (**c**) mammary tissues from 18-week-old mice are shown. Brown staining in **C** indicates Ki67^+^ cells, from which the percentage of Ki67^+^ cells from each group is graphed in **d**. All values are depicted in the graph as the mean ± S.E. (**p < 0.01)

Extensive mammographic density and development are preceding factors that are associated with increased breast cancer risk [38, 39]. To determine the impact of buformin diet on mammary morphogenesis, premalignant mammary glands were harvested from 18-week-old MMTV-erbB-2 mice for whole mount and histological analyses. As shown in Fig. 5a, the control mice exhibited a more dense and complex ductal network with increased side branching, as compared to the buformin-fed mice. These findings indicate that buformin effectively impedes mammary morphogenesis and produces remarkable histoarchitectural changes, which are further supported by H&E stained tissues (Fig. 5b). In the H&E stained mammary tissues from MMTV-erbB-2 mice, buformin substantially reduced the ductal thickness and altered the epithelial cell organization (Fig. 5b). Moreover, the percentage of proliferating cells was significantly reduced by buformin in the mammary tissues, as indicated by Ki67 IHC staining (Fig. 5c-d). Together, our data demonstrate that buformin induces morphogenic changes in the premalignant mammary glands, which indicate precursory histological phenotypes that are associated with impeded mammary tumor development.

### Buformin selectively inhibits stem cell populations and suppresses the self-renewal of MECs from MMTV-erbB-2 mice

**Fig. 6:**
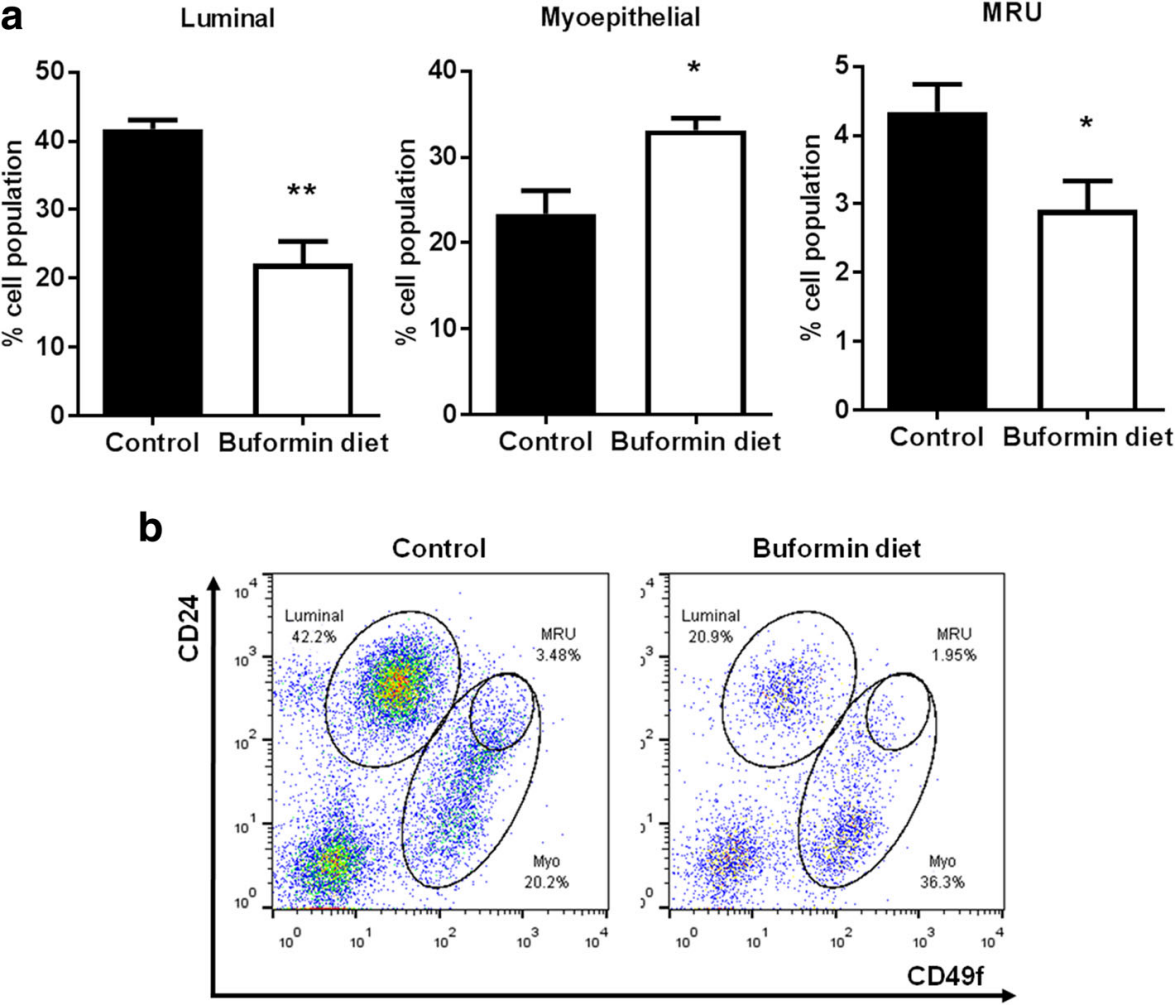
Buformin targets mammary stem cell populations in premalignant tissues from MMTV-erbB-2 mice. Primary MECs were isolated from 18-week-old mice that were fed control or buformin diets for 10 weeks (N = 4). Cells were labeled with fluorescent antibodies for flow cytometry analysis of different MEC populations. **a** The CD24/CD49f-probed cells produced 3 distinct cell populations, including luminal cells (left panel), myoepithelial cells (middle panel), and MRUs (right panel) that were compared between the control-fed and buformin-fed mice. Representative images of CD24/CD49f flow cytometry plots are shown in **b**. All values are depicted in the appropriate graphs as the mean ± S.E. (*p < 0.05, **p < 0.01)

Since mammary stem cells (MaSCs) give rise to proliferative luminal/progenitor cells and are precursors to CSCs, MaSCs play a critical role in mammary gland development and tumorigenesis [40, 41]. Therefore, we hypothesized that buformin may impair mammary morphogenesis and ultimately prevent tumorigenesis through MaSC reprogramming and the modulation of other stem cell populations in the premalignant mammary tissues. Recently, advances in stem cell technology have led to improved markers, including CD24, CD61, and CD49f cell surface markers, for individual cell populations. Using CD24/CD49f cell markers with flow cytometry, we were able to isolate 3 key subpopulations, luminal cells, basal/myoepithelial cells (Myo), and mammary reconstitution units (MRUs), found in primary MECs from MMTV-erbB-2 mice. Luminal cells are comprised of progenitor cells with proliferative properties, while the MRU population are enriched with MaSCs. The myoepithelial cell population represents basal cells. Fig. 6a indicates that the buformin diet significantly reduced luminal and MRU populations, while shifting towards the basal/myoepithelial cell population. Of note, buformin inhibits the overall lineage-negative cell population as displayed in fewer cells appearing in the representative flow cytometry plot (Fig. 6b).

**Fig. 7:**
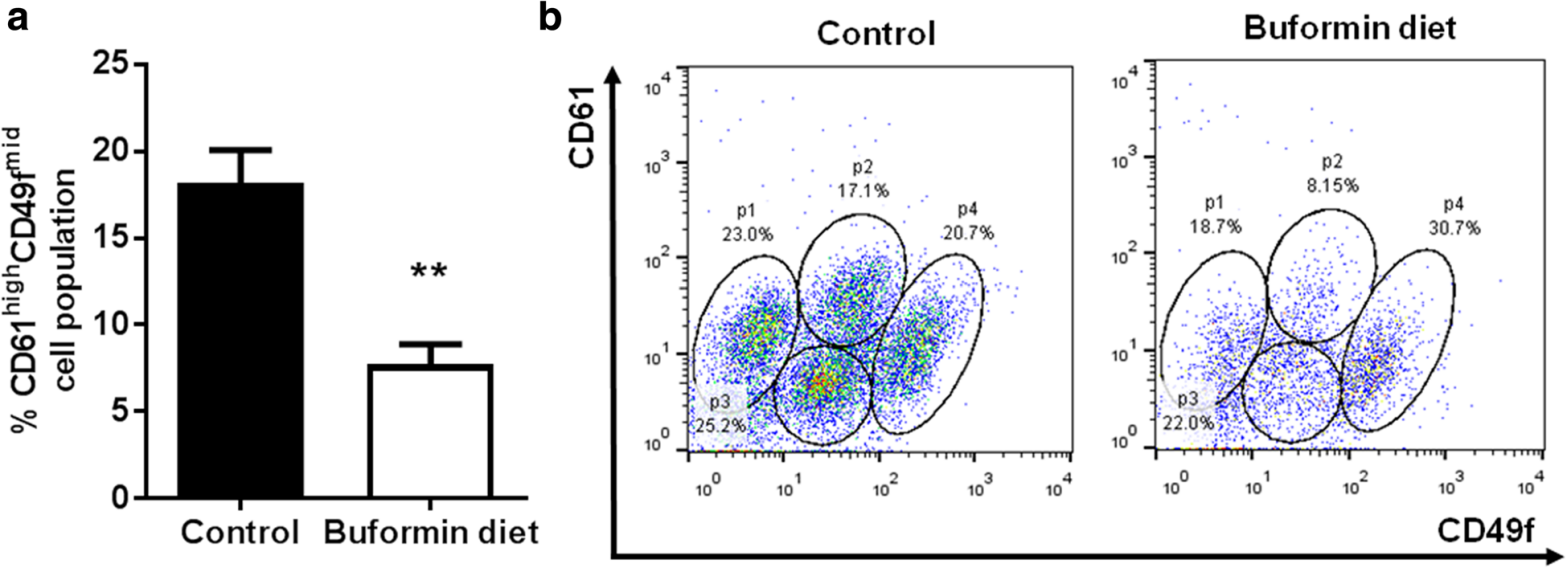
Buformin targets cancer stem cell/tumor-initiating cell populations in premalignant tissues from MMTV-erbB-2 mice. As detailed above, isolated MECs were labeled with CD61/CD49f markers and analyzed with flow cytometry (N = 4). **a** The percentage of CD61^high^CD49f^mid^ cell populations were compared between the control and buformin-fed mice with representative CD61/CD49f flow cytometry plots shown in **b**. All values are depicted in the graph as the mean ± S.E. (**p < 0.01)

**Fig. 8:**
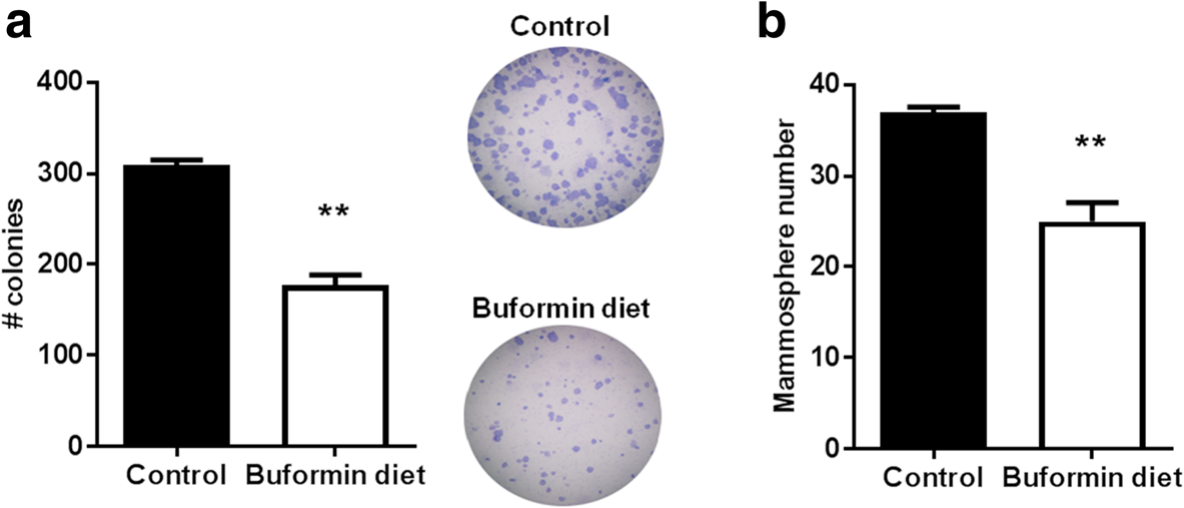
Buformin impairs the stemness of primary MECs from MMTV-erbB-2 mice. **a** Primary MECs were plated for a CFC assay (N = 3). After 7 days, the cells were fixed and stained as previously described. The number of colonies in the control and buformin diet samples was recorded. **b** Primary MECs were used for a primary mammosphere assay where the isolated cells were plated in ultra-low attachment plates for 7 days as described previously (N = 3). Then the primary spheres were counted. All values are depicted in the appropriate graphs as the mean ± S.E. (**p < 0.01)

According to the ‘CSC theory’, a cellular hierarchy exists where MaSCs can give rise to self-renewing TICs, which in turn promotes the initiation, progression, and metastasis of breast cancer [24, 42, 43]. To characterize the specific cell populations that contribute to tumorigenesis and are promising anti-cancer therapeutic targets, Lo *et al.* (2012) previously used CD61/CD49f cell surface markers to isolate these cell populations and reported that the CD61^high^/CD49f^mid^ population is comprised of CSCs/TICs in erbB-2-overexpressing mammary tumors [44]. Indeed, using CD61/CD49f cell markers, we demonstrated that buformin strikingly suppresses the CD61^high^CD49f^mid^ population (p2) of primary MECs from MMTV-erbB-2 mice (Fig. 7a-b). Since buformin only induced modest changes in the other CD61/CD49f cells populations, our data indicate that buformin selectively targets the CSC/TIC population for inhibition, which is consistent with our previous metformin report [13]. Based on our current findings, we further investigated the effects of buformin on the self-renewal property of stem cell subpopulations in premalignant MECs. Using a CFC assay, we determined in Fig. 8a that the buformin diet significantly diminished the colony formation as compared to the control diet. Results from the primary mammosphere formation assay reflect a similar trend from the CFC assay as the primary MECs from the buformin-fed mice formed significantly fewer spheres as compared to the control samples (Fig. 8b). The buformin-stimulated changes in CSC/TIC populations and stem-like properties of MECs indicate that buformin, like metformin, elicits its anti-cancer responses through selective targeting of CSCs/TICs *in vitro* (Fig. 3a) and *in vivo* (Fig. 7a-b).

### Buformin diet regulates mTOR, erbB-2/PI3K/Akt, ER, and Wnt/β-catenin signaling in mammary tissues from MMTV-erbB-2 mice

**Fig. 9:**
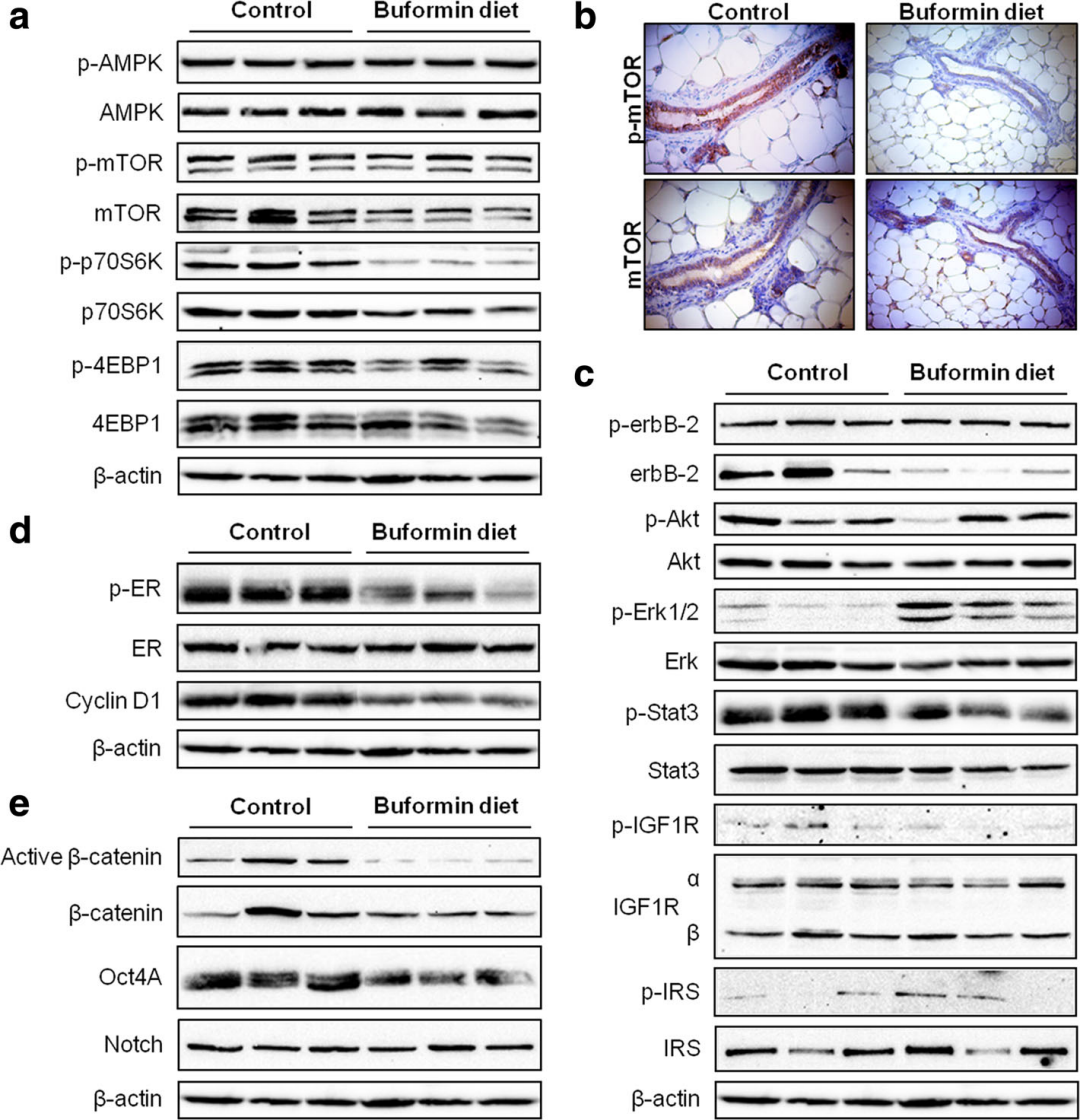
Buformin regulates signaling pathways that promote cell proliferation. Protein lysates were extracted from premalignant mammary tissues of 18-week-old MMTV-erbB-2 mice that were fed control or buformin diets for 10 weeks (N = 3). The expression and activation/phosphorylation of proteins associated with the mTOR (**a-b**), RTK (**c**), ER (**d**), and Wnt/β-catenin (**e**) pathways were examined using Western blot analysis (**a**, **c-e**) and IHC (**b**). Representative images of p-mTOR and mTOR immunostained mammary tissues from 18-week-old mice are shown in **b**

To uncover the underlying mechanisms that contribute to the buformin-induced changes in mammary gland morphogenesis and stem cell populations, we analyzed the expression of proteins involved in various signaling pathways. By analyzing the AMPK/mTOR pathway, we found that the activation/phosphorylation of downstream targets of mTOR, including p70S6K and 4EBP1, were downregulated by buformin diet (Fig. 9a). Interestingly, no significant changes in AMPK and mTOR activation/phosphorylation were observed in our Western blot results (Fig. 9a). Since discrepancies may exist between cell lysate-based and *in situ* analyses, we further examined the activation/phosphorylation of mTOR and AMPK in premalignant mammary tissues using IHC. We found that buformin diet does indeed suppress the activation/phosphorylation of mTOR, but does not activate AMPK (Fig. 9b, Additional file 1). Collectively, integrated data from Western blot and *in situ* analyses indicate an AMPK-independent mechanism of mTOR inhibition, which we will address in the *Discussion* section. Furthermore, the buformin diet induced an upregulation of phospho-Akt and downregulation of phospho-Erk and phospho-Stat3 in the MMTV-erbB-2 mice as compared to the control mice (Fig. 9c). This inverse response was consistent with the trend observed in our *in vitro* model (Fig. 4b). Additionally contributing to the anti-proliferative responses demonstrated in mammary tissues from buformin-fed mice, phospho-ER and Cyclin D1 were suppressed in relation to the control-fed mice (Fig. 9d). Wnt/β-catenin pathway results indicate the suppression of β-catenin activation, and Oct4A expression (Fig. 9e). Collectively, our signal transduction data implicate mTOR, RTK, ER, and Wnt/β-catenin signaling as major factors involved in the anti-proliferative mechanism of buformin that protects mammary tissues from tumor development *in vivo*.

## Discussion

Phenformin and buformin were first tested as anti-cancer agents around the 1970s and initially demonstrated promising cancer preventative and therapeutic responses. However, lactic acidosis, a consequence of lactate buildup from mitochondrial complex I inhibition, was a fatal side-effect associated with phenformin and buformin treatment in about half of the patients who developed this complication [45]. Unfortunately, the risk of harmful side-effects outweighed the clinical benefits and clinical trials investigating the anti-cancer effects of these drugs were terminated. In the past decade, renewed interest in biguanide agents as cancer preventative and treatment options have emerged due to the association of metformin with cancer prevention in Type II diabetes patients [3, 4]. To study the anti-cancer effects of buformin, while avoiding the occurrence of deleterious side-effects, our lab employs a method of drug administration where buformin is added to the standard diet of the subject animals. Our buformin diet was formulated based on the study by Zhu *et al.* (2015) that used a buformin diet at the same concentration (7.6 mmol buformin/kg of chow) with reported plasma (1.4 ± 1.8 μmol/L) and mammary carcinoma (6.7 ± 6.2 nmol/g) concentrations of buformin in their animal model of breast cancer [30]. Since both our study and the study by Zhu *et al.* used the same buformin concentration in the diet and demonstrated comparable tumor inhibition, we estimate that the plasma and tissue/tumor concentrations of buformin in our model should also be similar to what was previously reported. Importantly, the administration of buformin in the diet has successfully reduced the toxicity of buformin and increased the maximum tolerated dose in rodents [30]. Well-tolerated administration of buformin provides the basis for proof of concept studies that can be expanded for future research regarding the anti-cancer efficacy of buformin and other biguanides.

Previous reports on the anti-cancer efficacy of buformin have demonstrated overall inhibitory effects in endometrial cancer cell lines and *in vivo* carcinogen-induced mammary tumor models [30, 31]; nevertheless, the anti-cancer benefits specific to breast cancer and, in particular, different subtypes of breast cancer remain limited. As such, our aim to investigate the efficacy of buformin in various models of erbB-2-overexpressing breast cancer, which accounts for approximately 30% of human breast cancer cases [46], has provided further evidence of the anti-cancer potential of buformin. In our study, we report growth inhibition, targeted suppression of putative MaSC/CSC-enriched populations, and modulation of cell stemness, as measured by various analyses, in cellular, syngeneic, and transgenic models of erbB-2-overexpressing breast cancer. By evaluating the molecular responses to buformin treatment, we were able to propose several mechanisms that may contribute to the phenotypic changes that we report.

Our initial proof of concept experiments recapitulated the anti-proliferative effects of buformin that were previously determined by others in multiple cancer models [30, 31]. In our current study, we demonstrated that buformin inhibited cell growth and cell cycle progression, as analyzed by MTT, clonogenic, and cell cycle assays (Fig. 1). Buformin, when administered in the diet, also significantly impaired the growth of syngeneic tumors in MMTV-erbB-2 mice (Fig. 2). With the inhibition of major hallmarks of cancer *in vitro* and *in vivo*, we examined the effects of buformin in the premalignant mammary glands of MMTV-erbB-2 mice. Buformin treatment during the ‘risk window’ for spontaneous tumor development in these mice resulted in significant histoarchitectural changes, including decreased mammary ductal density and proliferative features in the preneoplastic mammary glands (Fig. 5). The mechanism by which buformin induces these anti-proliferative effects may involve several signal transduction pathways, including AMPK, RTK, and ER pathways, that are known to play critical roles in the regulation of cell proliferation.

AMPK, an energy sensor, regulates mTOR-mediated signaling involved in differentiation, transcription, and translation. In response to the mitochondrial dysfunction previously shown to be induced by buformin [47], we observed concomitant upregulation of p-AMPK and downregulation of p-mTOR and downstream targets, p-p70S6K and p-4EBP1, in erbB-2-overexpressing breast cancer cells *in vitro* (Fig. 4). Although the activation/phosphorylation of AMPK was not observed *in vivo* (Fig. 9a, Additional file 1), the evident suppression of mTOR, p70S6K, and 4EBP1 activation/phosphorylation (Fig. 9a-b) exhibited a similar trend to our *in vitro* signaling data and is indicative of buformin-stimulated alterations of an AMPK-independent mTOR signaling pathway. Several reports have similarly determined that metformin can inhibit mTOR signaling independent of AMPK activation in other *in vitro* and *in vivo* model systems [21, 48–51]. As such, REDD1 is an alternative mTOR regulator that has been reported to induce growth inhibition, cell cycle arrest, and apoptosis under nutrient deprived conditions, including in response to metformin treatment [21, 52]. Additionally, metformin has been shown to suppress mTOR activity through the upregulation of key components of the mTORC1 complex, PRAS40 and RAPTOR, in glioma cell and animal models [48]. Metformin has also been reported to suppress non-metastatic and metastatic canine mammary tumor cell lines *in vitro* via AMPK-dependent and -independent processes, respectively [49]. Importantly, mTOR is downstream of RTK signaling, including erbB-2 and IGF1R, which we have shown to be downregulated by buformin (Fig. 9). Further studies are warranted to fully understand the AMPK-independent mTOR inhibition by buformin that we have demonstrated in our *in vivo* mouse model*.*

Furthermore, previous studies have reported that biguanides negatively regulate insulin receptor (IR) and IR substrate (IRS), which result in IGF1R inactivation [53, 54]. As such, our data corroborate this concept as demonstrated by the decrease of IGF1Rα and p-IGF1R upon buformin treatment in the MMTV-erbB-2 mice, despite variations of IRS expression and phosphorylation between animals (Fig. 9). Importantly, IGF1R can form heterotrimers with multiple EGFR family members, including erbB-2 [55]. Metformin can disrupt the interaction of IGF1R and erbB-2 as well [27]. In our study, we demonstrate that buformin repressed erbB-2 activation alongside downstream inhibition of PI3K/Akt activity. Interestingly, buformin inversely modulated the phosphorylation of Akt and Erk1/2 in the SKBR3 cell line and MECs collected from MMTV-erbB-2 mice, but not BT474 cells (Fig. 4 and 9). Other reports using rapamycin, an mTOR inhibitor, on pancreatic cancer cells and metformin on NSCLC cells have also revealed differential effects on Akt and Erk activation, thus indicating a feedback mechanism involving mTOR and RTK signaling [56, 57]. Alternatively, the parallel decrease in p-Akt and p-Erk1/2, as we noted upon buformin treatment in BT474 cells, is supported by several recent reports, indicating that these inconsistencies in Akt and Erk activation may be cell-type specific [58].

Other signaling pathways, such as the ER pathway, regulate cellular growth and survival responses. In this context, we report that buformin inhibited ER activation/phosphorylation and downstream signaling of Cyclin D1 *in vivo* (Fig. 9). The regulation of these pathways is critical in erbB-2-overexpressing breast cancers due to IGF1R and/or erbB-2 crosstalk with ER [59, 60].

In addition to the inhibitory effects on mammary tumor growth, buformin also elicits substantial inhibition of cell populations associated with putative MaSCs (MRU population) and CSCs/TICs (ALDH^+^/CD61^high^CD49f^mid^ cells) in erbB-2-overexpressing breast cancer cell lines and primary MECs from preneoplastic mammary glands of MMTV-erbB-2 mice (Fig. 3, 6, and 7). Since MaSCs are integral for mammary gland development, mammary morphogenesis is also associated with MaSCs/CSCs. Suppressed MaSC and CSC populations (Fig. 6–7) are consistent with impeded mammary morphogenesis induced by buformin (Fig. 5). Importantly, the selective targeting of CSCs, as we show in our studies, is a promising preventative/therapeutic strategy to block pro-oncogenic events that contribute to cancer initiation in premalignant tissues and the progression of cancer at various stages.

The key to developing CSC/TIC inhibitors is to understand the underlying mechanisms that result in selective inhibition of CSC/TIC populations, as demonstrated by buformin in our models of erbB-2-overexpressing breast cancer. As a potentially critical pathway for the CSC-targeted effects of buformin, the Wnt/β-catenin pathway plays a substantial role in the regulation of numerous pro-cancerous cellular responses, including cell differentiation and proliferation [61, 62]. Our *in vivo* data indicates that buformin blocks the activation of β-catenin and other downstream signaling molecules (i.e. Oct4A) (Fig. 9), providing a potential connection between the differential epithelial subpopulations that we report in the buformin-treated samples as compared to the control-treated samples (Fig. 6–7). Due to the concurrent reduction in the CD61^high^CD49f^mid^ cell population and mammosphere formation efficiency in the MECs from buformin-treated mice, our results suggest that buformin may be inducing Wnt/β-catenin-mediated MaSC reprogramming to deter the differentiation into CSCs. This proposed mechanism would explain the selective targeting of CSCs by metformin, as previously published in our lab, and buformin [13].

## Conclusions

Taken together, we have demonstrated novel cancer inhibitory effects of buformin, especially on putative MaSCs/TICs, in premalignant mammary tissues. Our results also indicate that the erbB-2-overexpressing subtype of breast cancer may be more susceptible to the anti-cancer effects of buformin, due to the inhibition of numerous signaling molecules associated with RTK, especially erbB-2, signaling pathways. Our study has further clinical impact as demonstrated by the well-tolerated administration of buformin in the diet to avoid the previously reported toxic side effects, which improves the overall drug safety profile of buformin and paves the way for future preclinical and clinical investigation of buformin and other biguanides as cancer preventative and treatment options.

## Additional file

Additional file 1: Buformin does not upregulate AMPK activation in MMTV-erbB-2 mice. Representative images of p-AMPK immunostained mammary tissues from 18-week-old MMTV-erbB-2 mice that were fed control or buformin diets for 10 weeks are shown. (TIF 789 kb)

## Abbreviations

ALDH1: Aldehyde dehydrogenase 1
CFC: Colony-forming cell
CSC: Cancer stem cell
DAB: Diaminobenzidine
DEAB: Diethylaminobenzaldehyde
ER^+^: Estrogen receptor-positive
FACS: Fluorescence-activated cell sorting
FBS: Fetal bovine serum
FFPE: Formalin-fixed, paraffin-embedded
H&E: Hematoxylin and eosin
IHC: Immunohistochemistry
IR: Insulin receptor
IRS: Insulin receptor substrate
MaSCs: Mammary stem cells
MECs: Mammary epithelial cells
MMTV-erbB-2: FVB/N-Tg/MMTV-erbB-2
MNU: 1-methyl-1-nitrosourea
MRUs: Mammary reconstitution units
MTT: 3-(4,5-dimethythiazol-2-yl)-2,5-diphenyl- tetrazolium bromide
Myo: Myoepithelial cells
RTK: Receptor tyrosine kinase
t_1/2_: Half-life
TICs: Tumor-initiating cells
