# Supplementary figures and images for: Buformin inhibits the stemness of erbB-2-overexpressing breast cancer cells and premalignant mammary tissues of MMTV-erbB-2 transgenic mice
Source: J Exp Clin Cancer Res. 2017 Feb 13;36:28. doi: 10.1186/s13046-017-0498-0 (PMC5307817; doi:10.1186/s13046-017-0498-0)

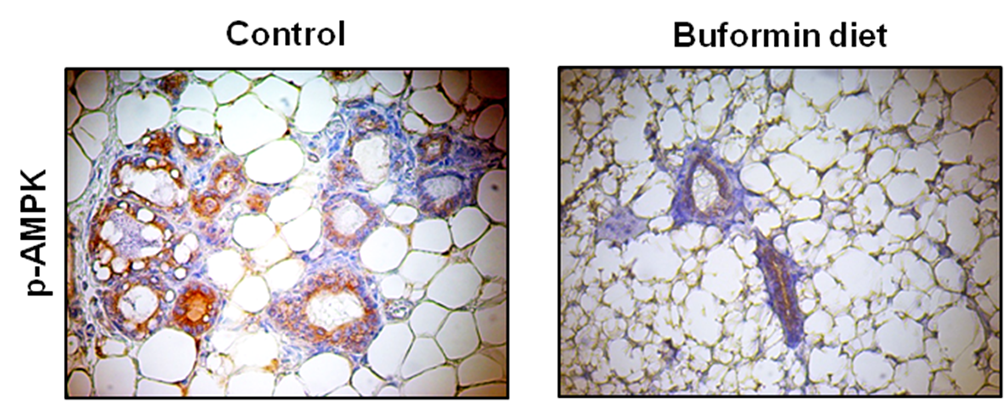

Supplement: Additional file 1: — Buformin does not upregulate AMPK activation in MMTV-erbB-2 mice. Representative images of p-AMPK immunostained mammary tissues from 18-week-old MMTV-erbB-2 mice that were fed control or buformin diets for 10 weeks are shown. (TIF 789 kb) [file 13046_2017_498_MOESM1_ESM.tif]
